# New Perspective on Digital Well-Being by Distinguishing Digital Competency From Dependency: Network Approach

**DOI:** 10.2196/70483

**Published:** 2025-03-25

**Authors:** Si Chen, Omid V Ebrahimi, Cecilia Cheng

**Affiliations:** 1 Department of Psychology University of Hong Kong Hong Kong China (Hong Kong); 2 Department of Experimental Psychology University of Oxford Oxford United Kingdom; 3 Department of Psychology University of Oslo Oslo Norway

**Keywords:** digital wellness, affective well-being, emotional regulation, coping, digital competence, digital autonomy, artificial intelligence

## Abstract

**Background:**

In the digital age, there is an emerging area of research focusing on digital well-being (DWB), yet conceptual frameworks of this novel construct are lacking. The current conceptualization either approaches the concept as the absence of digital ill-being, running the risk of pathologizing individual digital use, or follows the general subjective well-being framework, failing to highlight the complex digital nature at play.

**Objective:**

This preregistered study aimed to address this gap by using a network analysis, which examined the strength of the relationships among affective (digital stress and web-based hedonic well-being), cognitive (online intrinsic needs satisfaction), and social (online social connectedness and state empathy) dimensions of DWB and their associations with some major DWB protective and risk factors (ie, emotional regulation, nomophobia, digital literacy, self-control, problematic internet use, coping styles, and online risk exposure).

**Methods:**

The participants were 578 adults (mean age 38.7, SD 13.14 y; 277/578, 47.9% women) recruited from the United Kingdom and the United States who completed an online survey. Two network models were estimated. The first one assessed the relationships among multiple dimensions of DWB, and the second examined the relationships between DWB dimensions and related protective and risk factors.

**Results:**

The 2 resulting network structures demonstrated high stability, with the correlation stability coefficients being 0.67 for the first and 0.75 for the second regularized Gaussian graphical network models. The first network indicated that all DWB variables were positively related, except for digital stress, which was negatively correlated with the most central node—online intrinsic needs satisfaction. The second network revealed 2 distinct communities: *digital competency* and *digital dependency*. Emotional regulation emerged as the most central node with the highest bridge expected influence, positively associated with emotion-focused coping in the *digital competency* cluster and negatively associated with avoidant coping in the *digital dependency* cluster. In addition, some demographic differences were observed. Women scored higher on nomophobia (*χ*^2^_4_=10.7; *P*=.03) and emotion-focused coping (*χ*^2^_4_=14.9; *P*=.01), while men scored higher on digital literacy (*χ*^2^_4_=15.2; *P*=.01). Compared with their older counterparts, younger individuals scored lower on both emotional regulation (Spearman ρ=0.27; *P*<.001) and digital self-control (Spearman ρ=0.35; *P*<.001) and higher on both digital stress (Spearman ρ=−0.14; *P*<.001) and problematic internet use (Spearman ρ=−0.25; *P*<.001).

**Conclusions:**

The network analysis revealed how different aspects of DWB were interconnected, with the cognitive component being the most influential. Emotional regulation and adaptive coping strategies were pivotal in distinguishing digital competency from dependency.

## Introduction

### Background

Digital well-being (DWB), which refers to the subjective experience of optimal balance between the benefits and drawbacks obtained from online connectivity, has emerged as a critical area of concern in the current digital era [1]. As of July 2024, global estimates indicate that the number of active internet users has reached 5.45 billion, representing two-thirds of the world’s population, with a significant portion also engaging actively on social media platforms [2]. In this context, understanding and promoting DWB has become essential for bolstering individual well-being in an increasingly digitalized society.

Existing studies adopted 2 distinct approaches for conceptualizing this novel construct. The first approach conceptualizes DWB as the absence of problems resulting from internet and technology use, drawing parallels to frameworks of digital addiction (DA) [3-5]. This perspective encompasses various subtypes of problematic digital use, such as internet gaming disorder, smartphone addiction, and social media addiction, which have attracted growing attention from both the professional community and the general public [6-8]. DWB research that follows the DA framework has provided valuable insights into the risks of unhealthy digital behaviors [9-12]. However, equating well-being with the absence of such behaviors fundamentally differs from the broader conception of well-being as a state of “optimal psychological experience and functioning” [13]. An exclusive focus on ill-being can medicalize individuals’ relationships with digital devices, thereby overlooking the factors that foster positive and fulfilling digital experiences [1,14,15].

The second approach to conceptualizing DWB is grounded in subjective well-being (SWB) frameworks, emphasizing the hedonic and eudaimonic benefits of digital use [16,17]. This SWB approach to the conceptualization addresses affective well-being (eg, pleasure), satisfaction across various life domains (eg, interpersonal relations and work productivity), and overall satisfaction with contemporary digital life marked by constant internet connectivity and availability [17]. In contrast to the DA framework, the SWB approach highlights the concept of “controlled pleasure” and the use of digital devices to meet individuals’ intrinsic needs [18,19]. Despite the advantages of the SWB framework in promoting healthy digital relationships, its reliance on general SWB measures often fails to account for the domain specificity of digital connectivity [18,20].

### Toward a Tripartite Framework: Affective, Cognitive, and Social DWB

To address the shortcomings of both the DA and SWB frameworks, Büchi [17] proposed a new DWB framework that emphasizes how distal well-being outcomes (eg, psychological health and SWB) are related to 3 key constructs, namely, digital practices (eg, social media use and online behaviors), proximal harms (eg, digital stress), and benefits (eg, social connectedness and hedonic experience). These connections are further moderated by individual and situational variables [17]. Building on this theoretical understanding, this study expands the SWB framework by conceptualizing DWB as comprising individual dimensions of affective (ie, positive and negative affect), cognitive (ie, intrinsic needs satisfaction), and social (ie, experiences of connectedness and empathy) well-being, with an emphasis on the inherently digital nature of DWB [13,21].

In line with the conceptualization of affective well-being, the affective dimension of DWB reflects both positive and negative emotional experiences. On the negative side, digital stress encompasses the stress arising from the pressure of mobile connectivity, which can lead to expectations for constant availability (ie, availability stress) and feelings of being overwhelmed due to an excess of information on the web (ie, information overload) [22]. These affective stressors have been shown to have negative influences on mental health [22-24]. Conversely, the positive aspect of affective DWB includes the hedonic pleasure derived from engaging in digital activities [1,25].

A key conceptual component of DWB, aligned with the SWB framework, pertains to the cognitive dimension. Cognitive well-being refers to individuals’ evaluative judgments of their overall life satisfaction and satisfaction within specific domains [21]. Drawing from the SWB framework, previous DWB research has highlighted the importance of 3 core needs—autonomy, competence, and relatedness—in determining satisfaction with digital use [16]. This study focuses on intrinsic needs for autonomy and competence, which represent eudaimonic benefits derived from digital connectivity [1,16].

The increasingly pervasive role of social media in daily social interactions, a factor that has fundamentally transformed the processes of socialization, highlights the need to examine the social aspect as a distinct dimension of individual DWB. To unpack how individuals perceive and experience their relations with others and society at large through digital activities, this study operationalizes social DWB as comprising online social connectedness and online state empathy [26-30].

### Enhancing the DWB Framework: Role of Protective and Risk Factors

To formulate a comprehensive conceptual framework of DWB that addresses the variability inherent in digital connectivity [17], this research incorporates a cluster of DWB-related factors (ie, protective and risk factors).

One body of studies focusing on DWB enhancement highlighted several protective factors, including the ability to regulate one’s emotions (ie, emotional regulation), the ability to resolve conflicts between digital use and real-life goals (ie, digital self-control), skillful use of technologies (ie, digital literacy), and adaptive coping strategies such as emotion-focused coping [31-33]. Digital platforms provide users access to emotionally charged content and facilitate swift emotional exchanges through media emotional affordances such as likes, comments, and reactions [34,35]. Therefore, effective emotional regulation and adaptive emotion-focused coping are essential to prevent negative impacts from these interactions. Studies have indicated that individuals with stronger emotional regulation report better well-being and fewer problematic digital behaviors [36-38]. In addition to emotional affordances, online connectivity poses another threat to people’s DWB. Self-control becomes crucial for maintaining responsible internet use and resisting excessive use [4,39-41]. Studies also revealed the important role of digital literacy in a meaningful digital experience, which contributes to individual cognitive well-being by meeting their needs for competence and autonomy through skillful digital use [42,43].

Another body of DWB studies that follows the DA framework has identified several key affective and behavioral risk factors, including emotional attachment to mobile phones (ie, nomophobia), addictive internet use (ie, problematic internet use), maladaptive coping (ie, avoidant coping), and involuntary exposure to unwanted experiences (ie, online risk exposure) [10,44-46]. Meta-analyses revealed the harmful effect of overreliance on phone and internet use on mental health outcomes [47,48]. In addition, online risks, including unwanted explicit content, cyberbullying, sexual solicitation, and leaking of privacy, have been shown to elicit emotional and psychological harm, especially for younger internet users [46,49]. Avoidant coping refers to attempts to ignore or evade stressors rather than addressing them by attending to distractive digital activities. This maladaptive coping can reduce overall resilience and exacerbate feelings of stress, anxiety, and dissatisfaction [50].

In summary, this study examines the relationships among the affective, social, and cognitive dimensions of DWB as well as the associations between its related factors, which are a selected cluster of affective, cognitive, and behavioral protective and risk factors. This study uses a system-based approach [51] to uncover patterns and interactions that contribute to the overall structure of DWB. Using network analysis [52], it aims to reveal how various psychological and contextual factors (ie, related factors) relate to specific DWB components while accounting for the influence of other variables in the network. In doing so, this approach aims to provide a deeper understanding of the construct, illustrating how dimensions of DWB and its related factors collectively shape digital wellness.

## Methods

### Recruitment

This online study was accessible to participants using any type of digital device with internet access. Recruitment was facilitated through Prolific Academic, a crowdsourcing platform that offers access to large and diverse samples while ensuring high data quality for academic research [53]. Eligible participants included adults aged ≥18 years from the United Kingdom and the United States with a good track record on the survey platform (ie, an approval rate of ≥90% in previous surveys). Participants were informed about the study’s objectives, which involved completing a series of survey questions exploring their digital and online habits and activities. The hourly compensation rate of £2.50 (US $2.30) was provided upfront to ensure transparency and encourage voluntary participation. A total of 578 participants were recruited.

### Research Design and Procedure

This study used a cross-sectional design to collect data using self-report questionnaires administered through the Qualtrics software. Interested and eligible individuals were directed to a survey via a URL provided on the Prolific recruitment platform. Participants indicated their informed consent by checking a box before starting the survey. The survey included the measures described in the Overview section, which were presented in English and randomized in order. After completing the survey, participants were thanked and compensated.

### Measures

#### Overview

For each variable, the sum scores of the measures were incorporated as nodes in the network. Further details of the measures administered in the study are shown in Table 1.

**Table 1:** All measured variables included in the network analysis (N=578)^a^.

Label			Variable or node		Definition		Sample items
**Affective DWB^b^**							
	ADWB^c^1	Digital stress		The degree to which people felt stressed and pressured due to mobile communication and information overloads		“I feel stressed by the amount of contact through my mobile phone.” “I am pressured to respond quickly to all calls or texts.” “I am frequently overwhelmed by the amount of information available online.”	
	ADWB2	Web-based hedonic well-being		Hedonic experience from online activities		“Being online entertains and stimulates my mind.”	
**Cognitive DWB**							
	CDWB^d^	Intrinsic needs satisfaction		Individual satisfaction with needs for autonomy and competence resulted from the use of social media		“When I am on social media, I feel free to be who I am.” “When I am on social media, I feel very capable and effective.”	
**Social DWB**							
	SDWB^e^	Social digital well-being		The ability to understand, feel, and sympathize with others in digital space (online state empathy) and the degree to which people feel connected and related to others on the web (online social connectedness)		“I knew what the person I was interacting with felt emotionally.” “I felt the same way as the individual I was interacting through digital interactions.” “I feel close to people online.” “I don’t feel related to most people on social media.” [Reverse coded]	
**Affective**-**related factors**							
	AC^f^1	Emotional regulation		The ability to recognize and exert control over one’s own emotional state		“I have difficulty making sense out of my feelings.” [Reverse coded] “When I am upset, I have difficulty thinking about anything else.” [Reverse coded]	
	AC2	Nomophobia		The fear of being disconnected from the digital space		“If I did not have my smartphone, I would be uncomfortable because I could not stay up to date with social media and online networks.” “Running out of battery in my smartphone would scare me.”	
**Cognitive-related factors**							
	CC^g^1	Digital literacy		Individual ability of skillful digital use		“I know how to open a new tab in my browser.” “I feel comfortable deciding who to follow online (eg, on services like Twitter or Tumblr).”	
	CC2	Digital self-control		The ability to successfully manage goal conflicts between digital use and real-life activities		“How often do you give in to a desire to use social media even though your social media use at that particular moment makes you delay other things you want or need to do?” [Reverse coded]	
**Behavioral-related factors**							
	BC^h^1	Problematic internet use		Compulsive and excessive use of internet		“I lose track of time online.” “I feel lost if I can’t go online.”	
	BC2	Problem-focused coping		Coping by directly confronting a stressor in an attempt to decrease or eliminate it		“I’ve been thinking hard about what steps to take.”	
	BC3	Emotion-focused coping		Coping using skills for processing and dealing with feelings that arise due to stressful situations		“I’ve been making fun of the situation.”	
	BC4	Avoidant coping		Coping by not addressing the problem directly but instead disengaging from the situation		“I’ve been turning to work or other activities to take my mind off things.”	
	BC5	Online risk exposure		The frequency at which individuals are exposed to unwanted stressful events on the web		“Someone made rude or mean comments about you or threatened you in some way online.” “You saw online stories, images, or videos that contained excessive violence that made you feel uncomfortable.”	

^a^This table outlines all the variables tested in the network analysis, detailing their operational definitions and sample items. All the variables were measured using validated psychological scales in an online self-report survey conducted from June 2024 to July 2024.

^b^DWB: digital well-being.

^c^ADWB: affective digital well-being.

^d^CDWB: cognitive digital well-being.

^e^SDWB: social digital well-being.

^f^AC: affective-related factors.

^g^CC: cognitive-related factors.

^h^BC: behavioral-related factors.

#### Digital Stress

The Entrapment Scale [54] consists of 6 items that assess stress related to mobile communication and 3 items that evaluate information overload. Participants rated each item on a Likert scale ranging from 0 (strongly disagree) to 4 (strongly agree), indicating the extent to which they feel pressured by online communication and information available on the web. The sum score ranges from 0 to 36, with higher scores indicating greater levels of digital stress due to mobile entrapment and information overload.

#### Web-based Hedonic Well-Being

Web-based hedonic well-being was measured using the Online Hedonic Scale [55], which comprises 3 items that assess pleasant subjective experience with digital activities. Participants rated each item on a Likert scale ranging from 0 (strongly disagree) to 4 (strongly agree), indicating the degree to which they derive pleasure from being on the web. The sum score ranges from 0 to 12, and higher scores indicate greater levels of web-based hedonic well-being.

#### Intrinsic Needs Satisfaction

The 6-item Autonomy and Competence subscale was adapted from the Intrinsic Need Satisfaction Scale [16], which is a 12-item measure designed to assess individual intrinsic need satisfaction derived from digital use. Participants rated each item on a Likert scale ranging from 0 (strongly disagree) to 4 (strongly agree), reflecting the extent to which they feel their needs for autonomy and competence were fulfilled through social media engagement. The sum score ranges from 0 to 48, with higher scores indicating greater levels of internal need satisfaction.

#### Online Social Well-Being

The State Empathy Scale [56] is a measure consisting of 9 items that assess an individual’s ability to understand, feel, and empathize with others in the digital space. The online social connectedness measures were adapted from the Social Connectedness Scale [28], which also includes 9 items evaluating the extent to which individuals feel connected and related to others on social media. Participants rated each item on a Likert scale ranging from 0 (strongly disagree) to 4 (strongly agree), indicating the extent to which they experience empathy during online interactions. The sum score ranges from 0 to 72, with higher scores indicating greater levels of online social well-being.

#### Emotional Regulation

The Difficulties in Emotion Regulation Scale [57] consists of 16 items that assess individuals’ challenges in recognizing and managing their emotional states. Participants rated each item on a Likert scale ranging from 0 (strongly agree) to 4 (strongly disagree), reflecting the degree to which they experience difficulties in emotional regulation. The item scores are reversely coded, and the sum score ranges from 0 to 64, with higher scores reflecting greater levels of emotional regulation.

#### Nomophobia

The Nomophobia Scale [58] comprises 20 items that measure the fear of being unable to communicate and access information, losing connectedness, and sacrificing convenience due to disconnection from mobile phone connectivity. Participants rated each item on a Likert scale ranging from 0 (strongly disagree) to 4 (strongly agree), reflecting the extent to which they experience the aforementioned type of fear. The sum score ranges from 0 to 80, with higher scores indicating greater levels of nomophobia.

#### Digital Literacy

The Digital Literacy Scale [59] consists of 35 items that assess individuals’ ability in skillful digital use. Participants rated each item on a Likert scale ranging from 0 (strongly disagree) to 4 (strongly agree), reflecting their level of digital competence. The sum score ranges from 0 to 140, with higher scores reflecting greater levels of digital literacy.

#### Digital Self-Control

The Social Media Self-Control Failure Scale [60] consists of 3 items that evaluate self-control failures arising from goal conflicts between digital and real-life activities. Participants rated each item on a Likert scale ranging from 0 (strongly agree) to 4 (strongly disagree), reflecting the extent to which they struggle to manage goal conflicts between online engagement and real-life activity. The sum score ranges from 0 to 12, with higher scores indicating greater levels of digital self-control.

#### Problematic Internet Use

The Problematic Internet Usage Scale [8] consists of 15 items that measure compulsive and excessive internet use. Participants rated each item on a Likert scale ranging from 0 (strongly disagree) to 4 (strongly agree), indicating the extent of their problematic internet use. The sum score ranges from 0 to 60, with higher scores reflecting greater levels of problematic internet use.

#### Coping Styles

The Brief Coping Orientation to Problems Experienced Inventory [61] consists of 28 items that assess individual preferred coping styles (ie, problem focused, emotion focused, and avoidant). Participants rated each item on a Likert scale ranging from 0 (strongly disagree) to 4 (strongly agree), reflecting the extent to which they adopt a particular coping style. The sum score ranges from 0 to 32 for problem-focused coping, 0 to 48 for emotion-focused coping, and 0 to 32 for avoidant coping. Higher scores indicate greater deployment of the corresponding coping style.

#### Online Risk Exposure

The Online Risk Exposure Scale [46] consists of 16 items that measure the frequency of involuntary exposure to unwanted stressful events on the web. Participants rated each item on a Likert scale ranging from 0 (never) to 4 (always), indicating the frequency of their risk exposure to unwanted online activities. The sum score ranges from 0 to 64, with higher scores indicating greater frequencies of online risk exposure.

### Ethical Considerations

The study was preregistered on the Open Science Framework [62]. Ethics approval for this study was obtained from the Human Research Ethics Committee of the University of Hong Kong before the study began (EA240174). Before participation, individuals were presented with information regarding the study’s objectives, their rights to withdraw at any time without consequences, and the requirement for informed consent for research participation. All participants were compensated at a rate of £2.50 (US $2.30) per hour upon completing the study. To protect privacy and confidentiality, all data were anonymized and identified only through a randomly assigned participant ID. Responses were securely stored following data protection guidelines, including encrypting data during both storage and transmission.

### Statistical Analysis

All analyses were conducted using R software (version 4.1.3) and RStudio (R Core Team, 2024).

#### Preliminary Analysis

Shapiro-Wilk normality tests were first used to check normality. Kruskal-Wallis tests were conducted to examine potential differences across gender groups in cases of nonnormally distributed data. If significant differences (ie, *P*<.05) were detected, post hoc pairwise comparisons were performed using the Wilcoxon rank sum test with continuity correction to investigate differences across gender categories. The comparisons were adjusted using the Bonferroni correction method. Spearman rank-order correlation tests were conducted to assess differences based on age distribution.

#### Network Analysis

The R package *huge* was first used to apply nonparanormal data transformation for skewed data (version 1.3.5; Jiang et al [63]). Following guidelines for network analysis, variables were theoretically selected to avoid conceptual overlap [52,64]. A data-driven method was also applied to confirm this [65]. First, the correlation matrix was checked to ensure that it was positive definite, controlling for linear combinations among the variables. Then, the *goldbricker* function from the R package *networktools* (version 1.5.2; Jones [66]) was used to identify potential redundant variables. No redundancies were found, supporting that the theoretical selection had yielded nonoverlapping variables.

A regularized graphical Gaussian model (GGM) was used to estimate 2 network structures [67,68]. The first network (DWB network) contained the affective, cognitive, and social dimensions of DWB. The second network included DWB factors from the first network and related risk and protective factors selected from previous DWB research. The DWB-related associations were examined in the second network model (DWB-related network).

The network models were constructed using the EBICglasso algorithm in the R package *qgraph* (version 1.9.4; Epskamp et al [69]). This procedure involves using the graphical least absolute shrinkage and selection operator to estimate a GGM through a regularization technique. This method balances model complexity and sample size to select the best-fitting model based on the extended Bayesian information criterion, which includes a penalty term that discourages overfitting. The graphical least absolute shrinkage and selection operator applies L1-regularization, shrinking small edge weights to zero, which results in a simpler network that highlights the most important connections. After constructing a series of networks, the model with the lowest extended Bayesian information criterion is chosen to ensure interpretability and avoid overfitting [70]. Networks were then visualized using the Fruchterman-Reingold algorithm [71]. Thicker edges indicate stronger associations between nodes, with red edges representing negative and blue edges representing positive associations.

The strength centrality measure for each variable was obtained across the 2 networks. This index reflects the overall strength connectivity of a variable with other variables from the studied system. The node with the highest strength centrality is considered the most central or influential, as it has the most and strongest associations within the network, underscoring its overall importance in the system. In this study, the strength centrality index was used to identify the most influential variable in examining the construct of DWB.

#### Community Detection for DWB-Related Network

The *spin glass* algorithm was used to detect the community structure of the DWB-related network (Yang et al [72]) using the R package *igraph* (version 7.2.3; Csárdi [73]). The communities represent clusters of nodes, which demonstrate stronger connections with one another compared to nodes from other clusters. In addition to strength centrality measures, the bridge expected influence (BEI) of each node was computed to reflect each node’s overall strength of connectivity with other communities detected in the DWB-related network using the R package *networktools* (version 1.5.2 [74]). This BEI index reveals the nodes that act as bridges between the different communities in the network. The node with the highest BEI indicates the crucial role played by the variable in linking the other cluster. In this study, the BEI index allows for the understanding of the mechanisms underlying the relationship between the protective and risk-oriented aspects of digital health.

#### Network Accuracy and Stability

Nonparametric and case-dropping bootstrapping tests were used to assess the accuracy and stability of the resulting networks using the R package *bootnet* (version 1.6 [75]). The accuracy of edge weights was tested using nonparametric bootstrapping with 1000 iterations. The stability of strength centrality and BEI measures were tested using case-dropping subset bootstrapping with 1000 iterations. In this process, the correlation between the original centrality indices and those derived from smaller subsets was calculated, where up to 75% (434/578) of the participants were dropped. The correlation stability coefficients were then computed, showing the largest proportion of data that can be excluded while still maintaining a correlation ≥0.70 with the original centrality indices, with 95% confidence. A network is considered stable if the correlation stability coefficient is at least 0.25, with a preferred value >0.50 [76].

## Results

### Preliminary Results

Table 2 summarizes the demographic characteristics of participants of this study.

The descriptive statistics of DWB and its related factors are summarized in Table 3. The Shapiro-Wilk normality results indicated that data for all measurements, except problematic internet use, were nonnormally distributed (Multimedia Appendix 1). Gender was found to be a significant factor in nomophobia (*χ*^2^_4_=10.7; *P*=.03), digital literacy (*χ*^2^_4_=15.2; *P*=.01), and emotion-focused coping (*χ*^2^_4_=14.8; *P*=.01). Specifically, a significant difference was observed, with women (median 48.00, IQR 36.00-60.00) scoring significantly higher than men (median 43.00, IQR 28.35-57.65) on nomophobia (*P*=.02). In addition, women (median 22.00, IQR 19.50-24.50) also scored significantly higher than men (median 21.00, IQR 17.50-24.50) on emotion-focused coping (*P*=.01). Conversely, men (median 113.00, IQR 102.50-123.50) scored significantly higher than women (median 107.00, IQR 96.00-118.00) on digital literacy (*P*=.01).

The Spearman rank-order correlation test results are summarized in Table 4. Statistically significant positive relationships were found between age and intrinsic need satisfaction, emotional regulation, and digital self-control. Conversely, age was found to be negatively related to digital stress, nomophobia, problematic internet use, problem-focused coping, emotion-focused coping, and online risk exposure.

**Table 2:** Demographic characteristics of the sample (N=578)^a^.

Demographic variable			Values
Age (y), mean (SD)			38.70 (13.14)
**Gender, n (%)**			
	Women	277 (47.9)	
	Men	288 (49.8)	
	Nonbinary or third gender	10 (1.7)	
	Other	1 (0.2)	
	Prefer not to say	2 (0.3)	
**Race, n (%)**			
	Asian	33 (5.7)	
	Black or African American	48 (8.3)	
	Hispanic or Latino	8 (1.4)	
	Native American	2 (0.4)	
	White	467 (80.8)	
	Other	22 (3.8)	
**Education, n (%)**			
	Less than high school diploma	12 (2.1)	
	High school diploma	126 (21.8)	
	Higher diploma	91 (15.7)	
	Associate degree	23 (4)	
	Bachelor’s degree	214 (37)	
	Master’s degree	82 (14.2)	
	Professional degree	13 (2.2)	
	Doctoral degree	17 (2.9)	
**Marital status, n (%)**			
	Single	161 (27.8)	
	In a relationship	154 (26.6)	
	Married	235 (40.7)	
	Divorced	18 (3.1)	
	Widowed	6 (1)	
	Other	4 (0.7)	

^a^The table provides a detailed breakdown of the major demographic information of the participants who took part in the online self-report survey conducted between June 2024 and July 2024.

**Table 3:** Descriptive statistics of digital well-being–related measurements (N=578)^a^.

	Scores, mean (SD; range)	Scores, median (IQR)
Digital stress	18.70 (7.07; 1.00-40.00)	18.00 (13.50-22.50)
Web-based hedonic well-being	10.80 (2.43; 0.00-16.00)	11.00 (10.00-12.00)
Intrinsic needs satisfaction	14.90 (4.16; 2.00-24.00)	15.00 (12.00-18.00)
Social digital well-being	35.60 (9.06; 9.00-61.00)	35.00 (29.00-41.00)
Emotional regulation	35.80 (11.80; 4.00-64.00)	36.00 (27.00-45.00)
Nomophobia	43.20 (18.00; 0.00-80.00)	45.00 (31.25-58.75)
Digital literacy	108.00 (17.00; 35.00-138.00)	110.00 (98.50-121.50)
Digital self-control	6.20 (2.76; 0.00-12.00)	6.00 (4.00-8.00)
Problematic internet use	26.80 (8.79; 0.00-49.00)	27.00 (21.00-33.00)
Problem-focused coping	13.70 (8.44; 0.00-43.00)	12.00 (6.00-18.00)
Emotion-focused coping	20.70 (4.52; 4.00-31.00)	21.00 (18.00-24.00)
Avoidant coping	25.90 (5.44; 3.00-42.00)	26.00 (22.63-29.38)
Online risk exposure	13.00 (4.45; 1.00-30.00)	13.00 (10.00-16.00)

^a^This table summarizes the descriptive statistics of all variables assessed in the online self-report survey conducted between June 2024 and July 2024.

**Table 4:** Spearman rank-order correlation test results of age differences (N=578)^a^.

	Spearman rank-order correlation	*P* value
Digital stress	−0.138	<.001
Web-based hedonic well-being	−0.022	.59
Intrinsic needs satisfaction	0.088	.03
Social digital well-being	−0.081	.051
Emotional regulation	0.265	<.001
Nomophobia	−0.200	<.001
Digital literacy	−0.077	.07
Digital self-control	0.350	<.001
Problematic internet use	−0.249	<.001
Problem-focused coping	−0.373	<.001
Emotion-focused coping	0.042	.31
Avoidant coping	−0.204	<.001
Online risk exposure	−0.144	<.001

^a^This table shows the correlational test results between age and measured variables, highlighting age-related patterns in digital behaviors. Data were collected through an online survey conducted between June 2024 and July 2024.

### Network Structure of DWB Factors

#### Network Estimation

**Figure 1:**
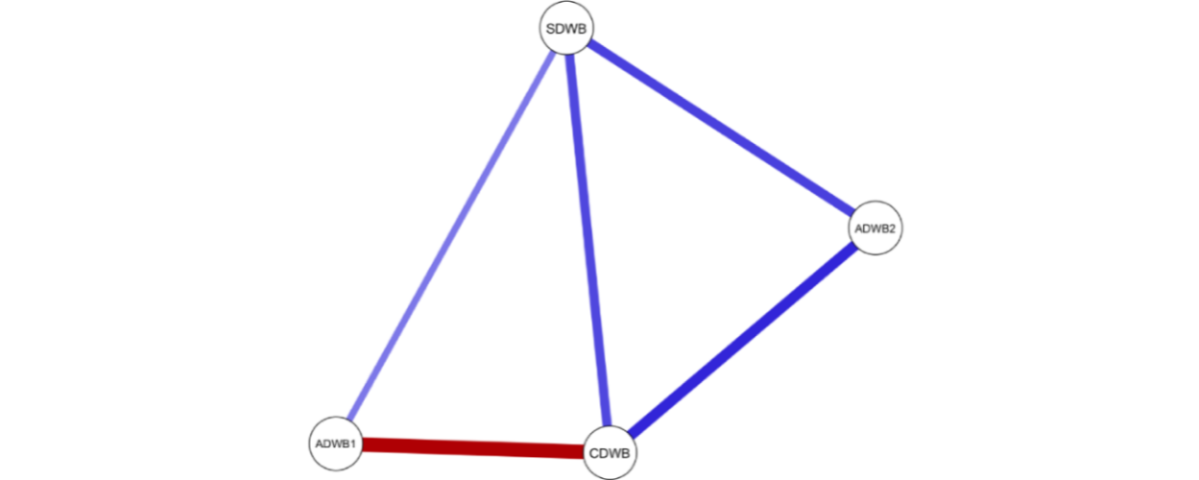
The digital well-being (DWB) network structure (N=578). The figure illustrates the relationships among 4 dimensions of DWB derived from a network analysis performed on survey data collected between June 2024 and July 2024. Blue edges indicate positive associations, while the red edge indicates a negative association between variables. The thickness of each edge reflects the strength of the observed relationships, with thicker edges denoting stronger associations. This DWB network highlights the interconnected nature of the DWB dimensions. ADWB1: digital stress; ADWB2: web-based hedonic well-being; CDWB: intrinsic needs satisfaction; SDWB: social digital well-being.

Figure 1 presents the regularized GGM network structure of the DWB measures. The well-being components were positively associated with each other, except for digital stress, which was negatively linked to intrinsic needs satisfaction (CDWB) and unrelated to web-based hedonic well-being (ADWB2). The strongest positive association was between CDWB and ADWB2. The second and third strongest positive edges were those between ADWB2 and social digital well-being (SDWB) and between intrinsic needs satisfaction (CDWB) and SDWB.

#### Strength Centrality

**Figure 2:**
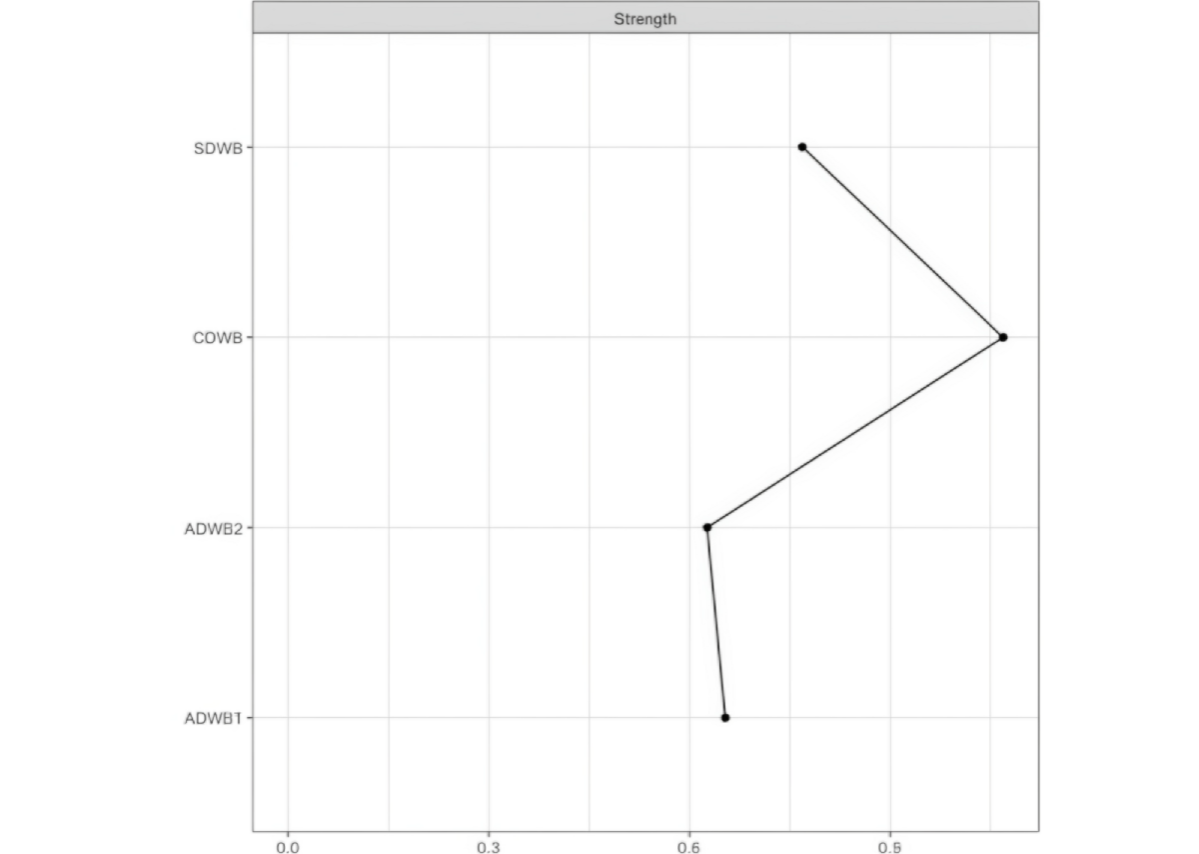
The centrality plot of the digital well-being network (N=578). This figure displays the centrality strength of all digital well-being variables derived from a network analysis performed on survey data collected between June 2024 and July 2024, highlighting their relative importance. The x-axis represents the centrality strength values, with higher values indicating more central roles in the network. ADWB1: digital stress; ADWB2: web-based hedonic well-being; CDWB: intrinsic needs satisfaction; SDWB: social digital well-being.

To examine the relative importance of each node in the DWB network, Figure 2 presents the strength centrality results. The cognitive component of DWB (ie, intrinsic needs satisfaction) had the highest strength centrality, followed by SDWB, suggesting the important roles played by the cognitive and social dimensions.

#### Network Stability

The correlation stability coefficient of strength centrality with the case-dropping subset bootstrapping method was 0.67, indicating that the centrality estimates were stable. Bootstrapping results for the estimated edge weights also provided support for the accuracy of edge weights in the resulting network (Multimedia Appendix 2).

### Network Structure of the DWB and Related Factors

#### Network Estimation

**Figure 3:**
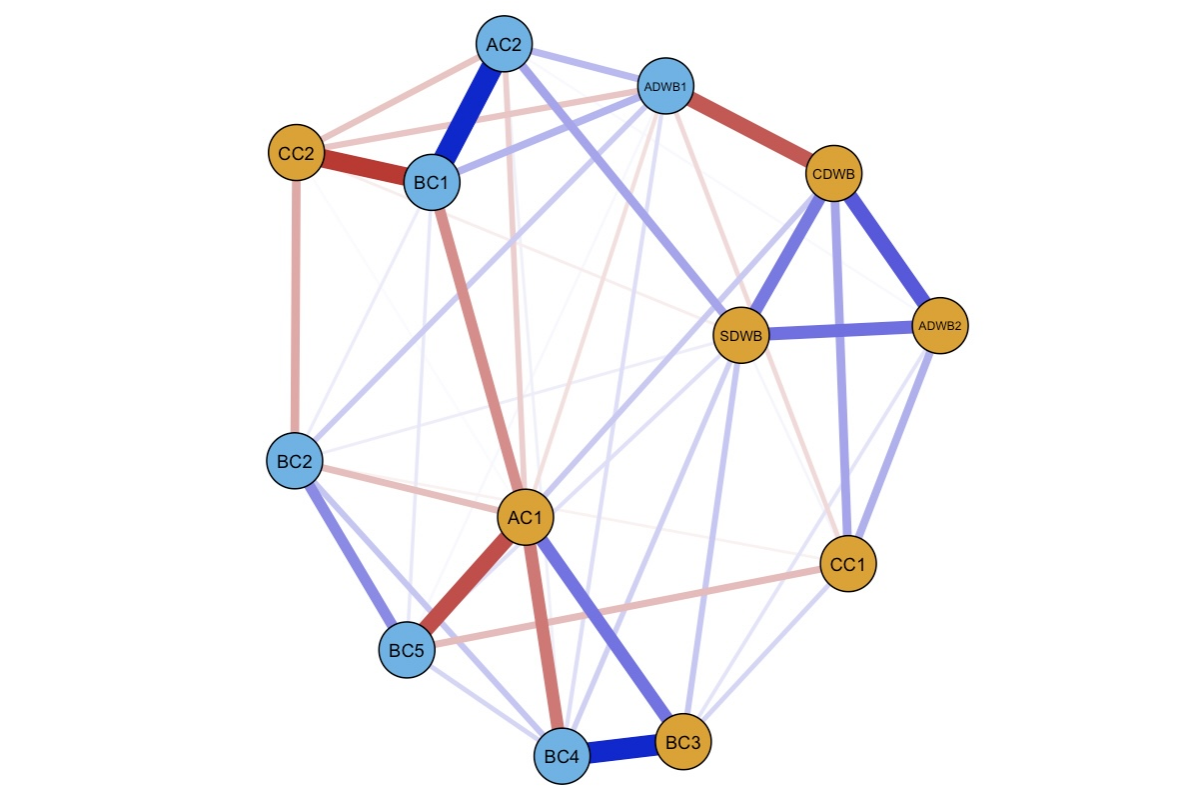
The digital well-being–related network structure (N=578). This figure illustrates the network structure connecting dimensions of digital well-being and associated protective and risk variables derived from a network analysis conducted on survey data collected between June 2024 and July 2024. The nodes represent specific variables, with orange nodes indicating the digital competency cluster and blue nodes representing the digital dependency cluster. Edges between nodes represent associations, with blue edges indicating positive relationships and red edges indicating negative relationships. The edges’ thickness corresponds to the associations’ strength, with thicker edges denoting stronger connections. AC2: nomophobia; ADWB1: digital stress; BC1: problematic internet use; BC2: problem-focused coping; BC3: emotion-focused coping; BC4: avoidant coping; BC5: web-based risk exposure; CC1: digital literacy; CC2: digital self-control.

Figure 3 presents the estimated structure of the DWB-related network. The spin glass community analysis detected 2 clusters. Consistent with the DWB network, the DWB factors were split into a *digital competency* (ie, healthy and effective use of technologies) community and a *digital dependency* (ie, harmful reliance on technologies) community. *Digital competency* included all DWB factors except digital stress (ADWB1) and affective and cognitive protective factors, including emotional regulation (AC1), digital literacy (CC1), and digital self-control (CC2). By contrast, negative affective DWB (ie, digital stress) was grouped into *digital dependency* with risk factors, including nomophobia (AC2), problematic internet use (BC1), problem-focused coping (BC2), avoidant coping (BC4), and the measure of participants’ past experiences with involuntary online risk exposure (BC5).

#### Strength Centrality

**Figure 4:**
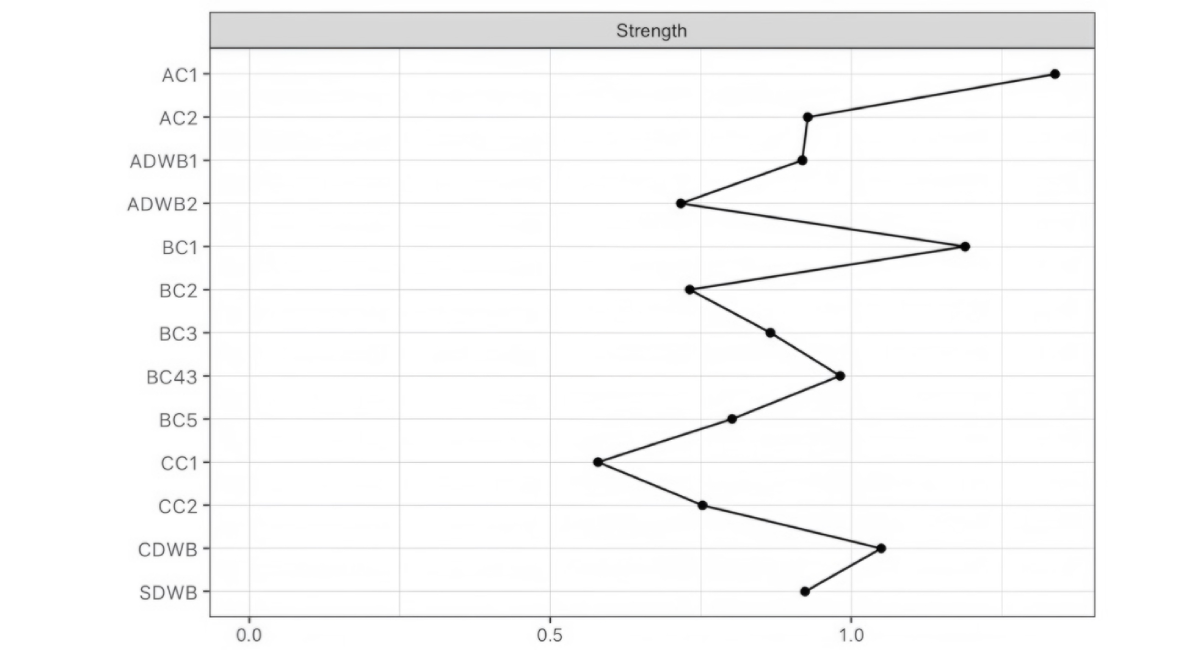
The strength centrality plot of the digital well-being–related network (N=578). The figure displays the strength centrality indices of each node derived from a network analysis conducted on survey data collected between June 2024 and July 2024. The x-axis represents the relative centrality strength values, with higher values indicating more central roles of the associated variables in the network. AC1: emotional regulation; AC2: nomophobia; ADWB1: digital stress; ADWB2: web-based hedonic well-being; BC1: problematic internet use; BC2: problem-focused coping; BC3: emotion-focused coping; BC4: avoidant coping; BC5: web-based risk exposure; CC1: digital literacy; CC2: digital self-control; CDWB: intrinsic needs satisfaction; SDWB: social digital well-being.

**Figure 5:**
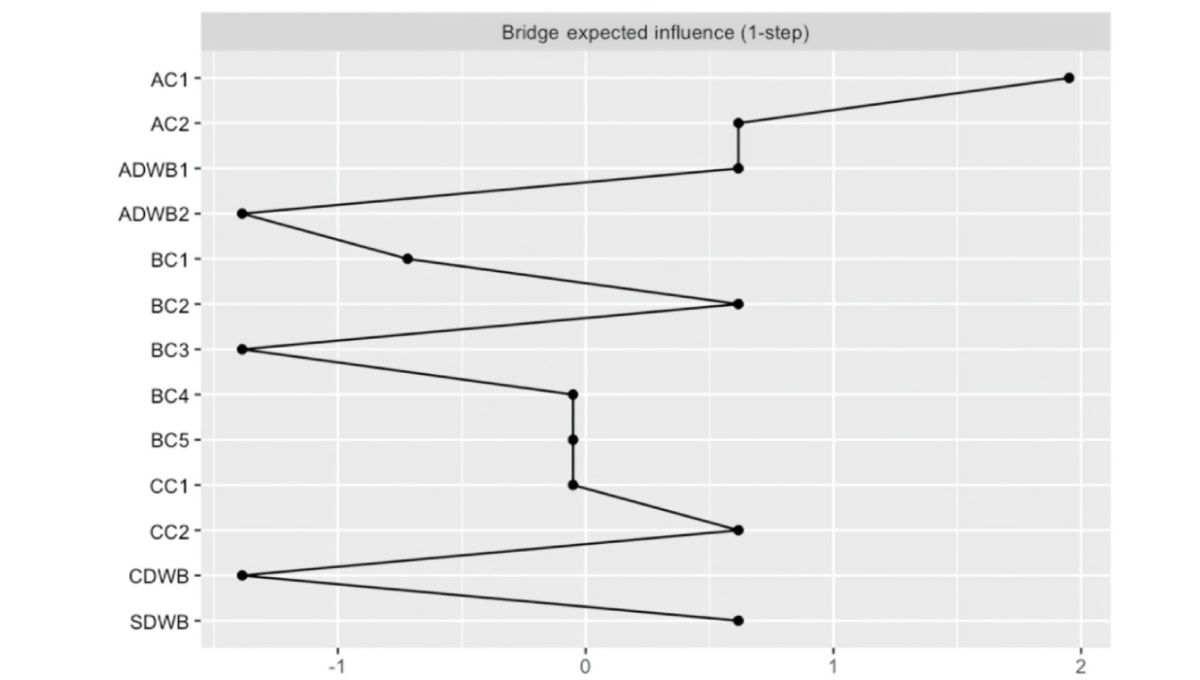
The bridge expected influence of the digital well-being–related network (N=578). The figure illustrates the bridge expected influence (1-step) values for each node in the digital well-being–related network derived from a network analysis conducted on survey data collected between June 2024 and July 2024. The y-axis lists the variables in the network, and the x-axis represents the relative bridge expected influence values. Higher values reflect nodes that act as critical bridging variables, connecting the digital competency and digital dependency clusters. AC1: emotional regulation; AC2: nomophobia; ADWB1: digital stress; ADWB2: web-based hedonic well-being; BC1: problematic internet use; BC2: problem−focused coping; BC3: emotion−focused coping; BC4: avoidant coping; BC5: web-based risk exposure; CC1: digital literacy; CC2: digital self-control; CDWB: intrinsic needs satisfaction; SDWB: social digital well-being.

Figures 4 and 5 present the strength centrality and the BEI measures for all DWB and related protective and risk factors. The nodes with the highest strength centrality indices were AC1, BC1, and CDWB. The most central nodes for each community were then examined. For *digital competency*, the most central factors were AC1 and CDWB. For *digital dependency*, BC1 and BC4 were identified with the highest centrality index.

#### Intercluster Connections

The importance of each node in connecting the identified communities within the network was examined with the BEI, as illustrated in Figure 5. The node with the highest BEI index was AC1, which played an important role in connecting the *digital dependency* and the *digital competency* cluster. Specifically, greater emotional regulation was positively associated with BC3 in the *digital competency* cluster. By contrast, greater emotional regulation was associated with lower BC5 and less BC4 from the *digital dependency* cluster.

The strongest positive links appeared between AC2 and BC1 in the *digital dependency* cluster and between CDWB and ADWB2 in the *digital competency* cluster. The strongest negative links were between CC2 and BC1, between AC1 and BC5, and between CDWB and ADWB1.

#### Network Stability

The correlation stability coefficients for strength centrality and BEI were both 0.75, implying a high stability of centrality and BEI indices in the generated DWB-related network. Bootstrapping results for the estimated edge weights also indicated accurate edge weights of the resulting network (Multimedia Appendix 3).

## Discussion

This study adopted a system-based framework to yield findings for conceptualizing the construct of DWB [51,64].

### Dimensions of DWB

The affective dimension comprises 2 relatively independent components: digital stress and web-based hedonic well-being (Figure 1). This finding contributes to the longstanding debates in affective well-being literature regarding whether positive and negative affect represent bipolar ends of a single construct or operate as independent dimensions [77-79]. Recent evidence supports that interactions between seemingly opposite affective dimensions can be context dependent [79]. In some contexts, people might experience both types of affect simultaneously (eg, during bittersweet experiences), while in other contexts, the presence of one might diminish the other [79].

Building on this line of work, our findings support the independent view of the affective well-being debate that digital stress and hedonic well-being appear relatively independent in digital contexts. The concept of a “stress paradox” on social network sites, where users simultaneously experience enjoyment and stress from factors such as social comparison and information overload, further speaks to our finding [80]. Together, these insights highlight the importance of examining the positive and negative affective dimensions independently, as they may coexist and impact DWB in unique ways.

Indeed, the positive and negative affective DWB showed opposite associations with the most central component of DWB, namely, cognitive DWB. Precisely, online intrinsic needs satisfaction was positively correlated with hedonic well-being and negatively correlated with digital stress. These 2 associations also represented the strongest edges within the DWB network. Such observations align with the self-determination theory, which posits that optimal well-being results from the fulfillment of individual psychological needs for competence, autonomy, and relatedness [81-83]. This study further distinguishes the cognitive needs for competence and autonomy, which directly address the eudaimonic benefits of digital connectivity, from the social needs for relatedness. Consequently, the findings resonate with psychological well-being literature, underscoring that digital eudaimonic experiences (ie, using digital devices to feel competent and autonomous) could play a crucial role in cultivating healthy digital relationships and minimizing potential harm from digital use [83,84].

The social dimension of DWB, encompassing digital empathy and online social connectedness, emerged as the second most central node in the network. It showed positive relations with all the other variables, albeit less strongly with digital stress, which was more closely related to the lack of perceived autonomy and competence. Research has shown that digital platforms not only allow people to extend their social relations but also provide avenues to maintain existing social networks [85,86]. Hence, our findings on the positive associations between the social dimension and the positive affective and cognitive components of DWB emphasize the pivotal role played by online experiences of empathy and connectedness in cultivating a satisfying and pleasurable digital relationship.

These findings have practical implications for intervention designs to leverage digital hedonic and eudaimonic experiences, an area of growing interest with artificial intelligence (AI) advancements. For instance, DWB programs can incorporate AI-driven tools to facilitate online intrinsic needs satisfaction with goal-tracking interfaces and adaptive feedback systems. These features can provide users with a tailored online experience and allow for monitoring of one’s progress, thus satisfying individual needs for competence and autonomy [87]. Moreover, AI-powered interventions could strengthen digital empathy and connectedness by analyzing and responding to online interactions to promote interpersonal understanding, which used to be a key challenge in online interactions due to the lack of nonverbal cues [88]. For example, social platforms could benefit from their large database to train AI to identify opportunities for meaningful engagement, such as recommending empathetic responses or fostering inclusive discussions.

Despite the benefits, caution should also be practiced in designing interventions that incorporate AI-driven tools, as the effectiveness of human-AI collaboration depends heavily on the relative strengths of each contributor [89]. While AI can greatly enhance users’ content creation and analytics strength by automating repetitive tasks, relying on AI for decision-making tasks may undermine human judgment due to the lack of contextual understanding. Therefore, interventions must carefully consider the context in which AI tools are deployed, ensuring that they complement rather than replace human agency. The importance of this balance is further discussed in the following section on digital competency and dependency.

### Emotional Regulation in Differentiating Competency From Dependency

To further understand the protective and risk factors associated with positive and negative dimensions of DWB identified in the initial network (Figure 1), the DWB-related network (Figure 3) revealed 2 distinct communities: *digital competency* and *digital dependency*. *Digital competency* refers to the effective integration and responsible use of technologies, such as successful self-control and skillful use, that can enhance one’s ability to achieve personal and professional goals while fostering optimal health outcomes [90,91]. By contrast, *digital dependency* represents a reliance on digital technologies that leads to undesirable psychological consequences, such as problematic internet use characterized by excessive or compulsive use of digital media [92,93]. These 2 distinct communities encapsulated the protective and risk-oriented dimensions of DWB, highlighting the dual nature of digital engagement, that is, its potential for both benefits and harm.

The affective aspect played a key role in fostering competency. Specifically, emotional regulation emerged as the most central actor in the network, displaying a positive link with emotion-focused coping from the *digital competency* cluster and a negative link with avoidant coping from the *digital dependency* cluster. This pattern aligns with previous coping literature that revealed a positive connection between successful emotional regulation and emotion approach coping [94]. Emotion-focused coping involves the conscious regulation of emotions to alleviate distress without necessarily resolving the underlying issue [94]. Conversely, avoidant coping, which emerged as the second most central node in the *digital dependency* cluster, involves evasion of both the stressor and the emotional response. It often consists of suppression, distraction, and withdrawal from one’s negative emotions, which leads to reduced well-being outcomes [95,96].

In the context of digital connectivity, a common motivation for digital use is to escape from boredom or real-life adversities, which is generally considered negative media consumption [97,98]. However, recent research on media escapism demonstrated that emotion approach coping coupled with cognitive control and self-efficacy can result in positive well-being outcomes [99,100]. Our findings further support this finding by emphasizing the role of emotional regulation in bridging the affective and behavioral coping aspects of DWB, thereby differentiating *digital competency* from *digital dependency*.

Within the *digital competency* cluster, the strongest positive association was between the satisfaction of individual needs for autonomy and competence and hedonic well-being. This finding aligns with the definition of DWB by Vanden Abeele [1] as an experience culminating in “controlled pleasure.” The issue of user autonomy has gained increasing significance. Amid the evolution of intricate algorithms, user autonomy is constantly shaped, constrained, and enabled by different dimensions of human-algorithm relations [101]. Consequently, individual decision-making, actions, and consequent well-being outcomes are guided by their knowledge, perception, and critical evaluation of omnipresent algorithmic outputs [101]. A recent study examining user well-being in online retail settings showed that perceived individual autonomy can predict hedonic enjoyment when engaging in both personalized purchase experiences and general browsing [102], supporting the observed link between autonomy need satisfaction and hedonic well-being.

In addition to digital autonomy, digital competence is another key conceptual component of DWB (eg, the European Commission’s Digital Competence Framework) [103]. Notably, research conducted during the COVID-19 pandemic revealed digital competence as a significant protective factor for general well-being [104]. Recent studies further advocated the benefits of digital competence across diverse age groups [105-108]. These findings underscore the importance of fulfilling individuals’ competence needs in both digital and offline spaces to successfully navigate their daily lives with pervasive technology presence.

The strongest positive association in the *digital dependency* cluster was between nomophobia and problematic internet use, both ascribing to an unhealthy attachment to digital connectivity. This finding aligns with DWB studies that use the DA framework [109,110]. One possible explanation for this positive association could be their shared dependence on mobile devices for connectivity, emotional support, social interaction, and information. Both problematic internet use and nomophobia involve the fear of disconnection and an unhealthy reliance on the internet, creating a cycle of compulsive phone and internet use.

Interestingly, this study revealed a stronger negative link between digital self-control and problematic internet use, while nomophobia displayed a positive, albeit weaker, association with social DWB from the digital competency cluster. The former finding is consistent with most DA literature that identifies self-control failures as the primary predictor for problematic internet use [32,40,111]. By contrast, the observed positive relationship between nomophobia and social well-being points to the need for a critical examination of frequent digital use. Research indicates that nomophobia is often linked to increased smartphone use [39,112]. As tools for social connection, smartphones enable individuals to maintain constant contact with their social networks, both on an interpersonal and societal level. This reliance on smartphones for communication may enhance empathy by fostering emotional exchanges and social feedback loops. Consequently, individuals higher in nomophobia also obtain more opportunities to experience and respond to others’ emotional states, thus reinforcing their sense of empathy through online engagement.

However, metacognitive evidence suggests that nomophobia may be related to difficulties in recognizing and expressing emotions and reduced face-to-face empathy [113-115]. Thus, while increased smartphone use may contribute to the observed positive association between nomophobia and social DWB, further studies are needed to understand how experiences of empathy and social connectedness differ in online and offline spaces.

Earlier, we discussed fostering DWB by satisfying users’ needs for competence and autonomy using AI-driven tools. However, there is a risk of AI technologies taking over users’ agency, thus leading to dependency. Here, the findings of the DWB-related network highlight the roles of emotional regulation and adaptive coping in distinguishing competency from dependency, thus offering several practical avenues for interventions aimed at reducing excessive use of technologies.

Meta-analytical findings support the effectiveness of digital gaming interventions in fostering adaptive emotional regulation [116,117]. Thus, integrating similar gamified features within digital social platforms could encourage users to identify and regulate their emotions during online interactions. For instance, social media platforms could introduce features such as interactive modules consisting of scenarios (eg, receiving negative feedback on social media), where users are guided through emotional regulation steps to interpret the situation and flexibly cope with the digital stressors presented to them.

In addition to general programs designed to cultivate individuals’ emotional regulation skills, tailored interventions can be implemented on digital platforms via emotion-sensitive algorithms to further enhance users’ abilities to regulate and cope with potential stressors arising from digital engagement. Excessive screen time or patterns of negative interactions, for example, can be identified to trigger break reminders or suggestions for positive content [118]. Such strategies will not only allow individuals to regulate their emotions but can also give rise to optimal DWB based on our findings.

### Gender and Age Differences

In addition to network findings on DWB and its related factors, statistically significant differences were found across age groups, suggesting that younger individuals may be more susceptible to unhealthy online behaviors, including problematic network use, difficulties in maintaining digital self-control, and heightened vulnerability to online risks [44,119]. These findings underscore the need for early onset of DWB interventions. School-based digital literacy programs and interactive workshops can teach students how to navigate digital spaces responsibly, develop appropriate self-control strategies, and critically assess online content. Such interventions should also be tailored to address the developmental needs of adolescents and young adults, equipping them with the tools to balance digital engagement with offline activities and develop healthier long-term digital habits.

Consistent with previous literature on digital literacy, men scored significantly higher than women on digital literacy, implying potential barriers that may prevent women from fully capitalizing on the advantages of digital technologies [120]. Conversely, women scored higher than men on nomophobia, which suggested escalating health concerns faced by women [121]. Interventions to bridge this gap could include gender-focused digital education programs and mentorship initiatives aimed at empowering women in digital spaces, emphasizing skill development in areas such as cybersecurity, data literacy, and content creation. In addition, tailored resources can be created to address the specific challenges women face, such as overreliance on digital connectivity and emotional susceptibility to online interactions.

### Limitations and Future Directions

Several limitations should be considered when interpreting the results of this study. One limitation is that the network analysis adopted a cross-sectional design, which presents methodological issues regarding the directionality and temporality of the results. Thus, longitudinal studies with multiple time points are needed to examine temporal changes in the variables included in the networks, as well as how the current networks relate to long-term well-being changes.

Another limitation is that the DWB networks mainly investigated psychological factors at the individual level. Given that DWB is a dynamic construct that emerges from the interplay between users and external digital factors, the influence of external factors should also be investigated [1,17]. For instance, algorithms often prioritize content that maximizes engagement with emotionally charged and polarizing posts, which can inadvertently amplify stress, anxiety, and feelings of inadequacy among users [122]. Similarly, interface nudges, such as the infinite scrolling design of TikTok, can prolong user engagement, making it harder for individuals to disengage and manage their screen time effectively [123]. To capture these dynamics, future efforts should broaden the current conceptualization of DWB by scrutinizing the interactions between psychological and external digital factors, such as unveiling the mechanisms underlying media affordances that serve to fulfill diverse motivational needs among users [35].

In addition to the study design, the sample of this study comprising adults from the United States and the United Kingdom raised issues on the generalizability of findings. The 2023 United Nations’ report on information society highlighted a growing gap between digital coverage and technological development between low-income (eg, South Africa) and high-income (eg, North America and Northern Europe) countries [124]. This disparity underscores the importance of contextualizing digital inequality, as it can significantly influence individuals’ digital health. For example, residents from countries with limited internet infrastructure may lack access to digital literacy initiatives, which are vital for fostering meaningful online experiences. Besides digital inequality, cultural differences in technology adoption can lead to varying effects of digital use on social well-being outcomes [125,126]. For example, higher self-disclosure has been associated with greater perceived social support and connection in Western cultures, where openness and self-promotion are valued [127]. In contrast, in cultures that prioritize modesty, increased online self-disclosure may lead to discomfort or stress in navigating different digital norms [128]. Therefore, our findings consisting mainly of White participants from Western high-income countries may not fully apply to countries with differing digital landscapes. More large-scale multinational studies on DWB should be conducted to increase the global applicability of our findings.

### Conclusions

Despite certain limitations, to the best of our knowledge, this is the first study to empirically conceptualize DWB and its protective and risk factors through a system-based approach. This research found that younger individuals face greater risks of problematic online behaviors, while gender differences in digital literacy and nomophobia indicate potential obstacles and health risks associated with digital use.

The conceptual network findings on various DWB factors suggest that digital stress and hedonic well-being are independent constructs of the affective dimension of DWB. Consistent with SWB and psychological well-being literature, the most central aspect of optimal DWB concerns fulfilling individuals’ psychological needs for competence and autonomy through digital eudaimonic benefits. By contrast, the hedonic experience was closely related to the social dimension of DWB. The network results of DWB and its related factors showed that emotional regulation and adaptive coping are key in distinguishing between digital competency and dependency, emphasizing the role of emotional regulation and cognitive control in fostering positive digital experiences. These insights highlight the importance of promoting autonomous and skilled digital use across demographics to ensure healthier digital engagement.

## Appendix

Multimedia Appendix 1 Nonparametric transformation for the skewed data for digital well-being and related variables.

Multimedia Appendix 2 Bootstrapping results for the estimated edge weights, edge difference, and node strength difference for the digital well-being network.

Multimedia Appendix 3 Bootstrapping results for the estimated edge weights, edge difference, and node strength difference for the digital well-being–related network.

## Abbreviations

AI: artificial intelligence
DA: digital addiction
GGM: graphical Gaussian model
SWB: subjective well-being

## Data Availability

The datasets generated and analyzed during this study are available on the Open Science Framework repository [62]. This repository includes the anonymized dataset included in the analysis. For further information regarding the data and statistical analyses, please contact the first author (SC).
