# Supplementary figures and images for: New Perspective on Digital Well-Being by Distinguishing Digital Competency From Dependency: Network Approach
Source: J Med Internet Res. 2025 Mar 25;27:e70483. doi: 10.2196/70483 (PMC11979542; doi:10.2196/70483)

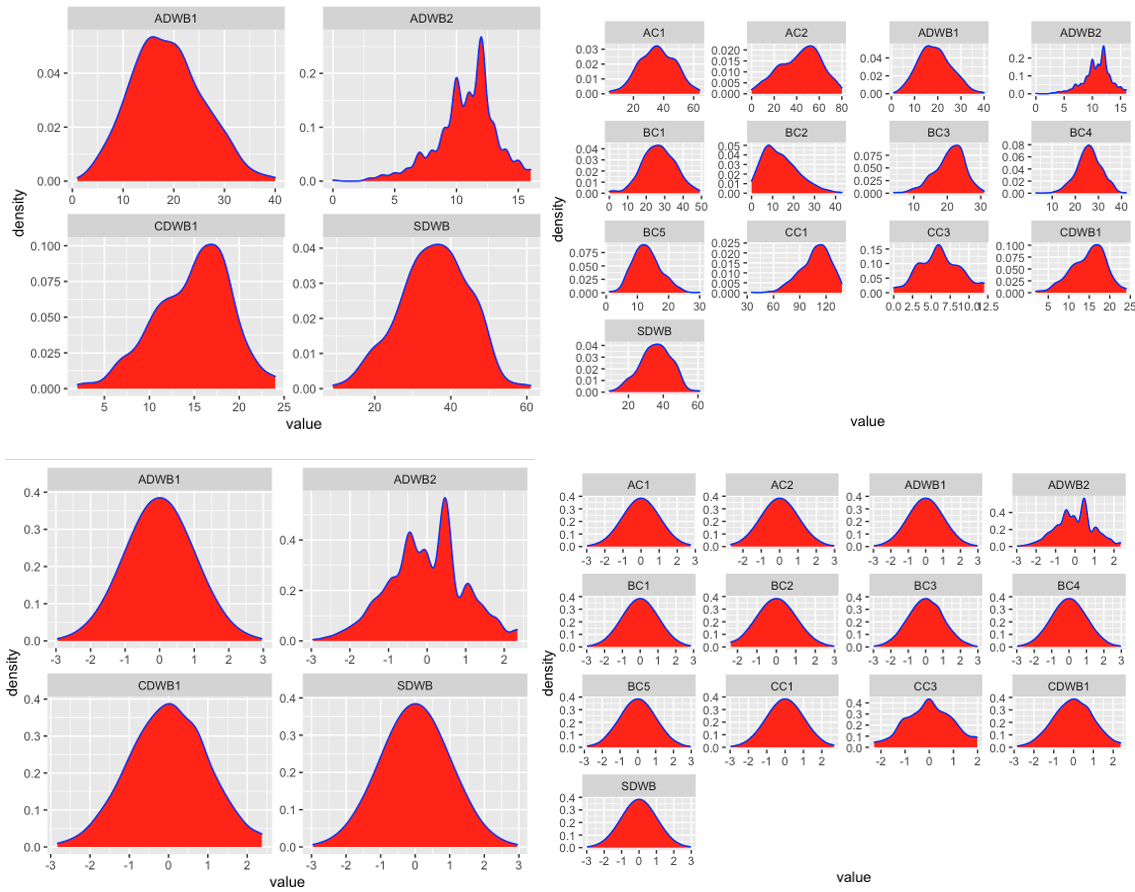

Supplement: Multimedia Appendix 1 [file jmir_v27i1e70483_app1.png]

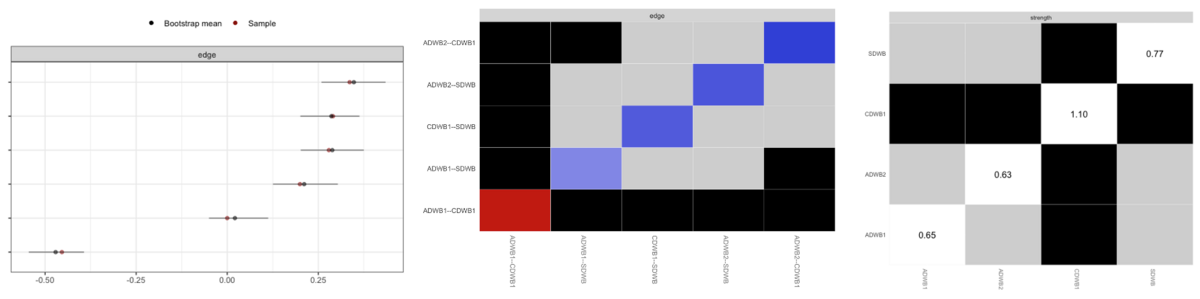

Supplement: Multimedia Appendix 2 [file jmir_v27i1e70483_app2.png]

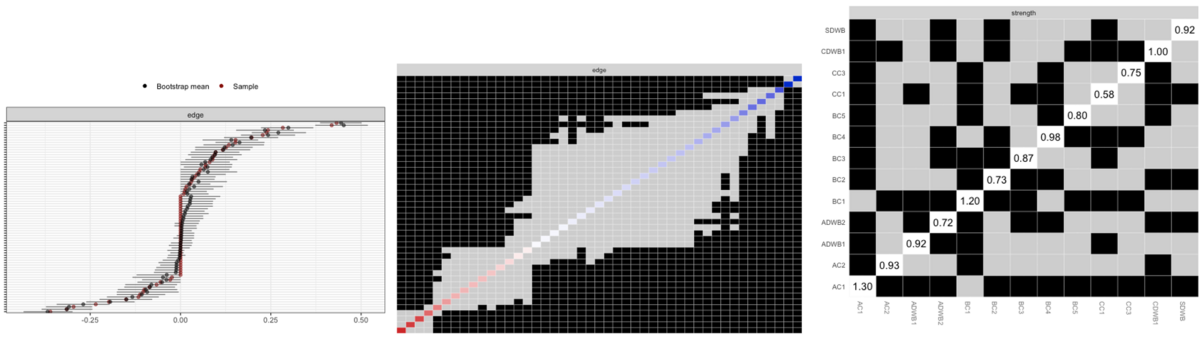

Supplement: Multimedia Appendix 3 [file jmir_v27i1e70483_app3.png]
